# An Exchange Mechanism for the Magnetic Behavior of Er^3+^ Complexes

**DOI:** 10.3390/molecules26164922

**Published:** 2021-08-13

**Authors:** Miroslav Georgiev, Hassan Chamati

**Affiliations:** Institute of Solid State Physics, Bulgarian Academy of Sciences, Tsarigradsko Chaussée 72, 1784 Sofia, Bulgaria

**Keywords:** single ion magnets, molecular magnets, spin Hamiltonian, post-Hartree–Fock method

## Abstract

We study the magnetic properties of the erbium based compounds, Na${}_{9}$[Er(W${}_{5}$O${}_{18}$)${}_{2}$] and [(Pc)Er{Pc{N(C${}_{4}$H${}_{9}$)${}_{2}$}${}_{8}$}]${}^{\cdot /-}$, in the framework of an effective spin exchange model involving delocalized electrons occupying molecular orbitals. The calculations successfully reproduce the experimental data available in the literature for the magnetic spectrum, magnetization and molar susceptibility in dc and ac fields. Owing to their similar molecular geometry, the compounds’ magnetic behaviors are interpreted in terms of the same set of active orbitals and thus the same effective spin coupling scheme. For all three complexes, the model predicts a prompt change in the ground state from a Kramer’s doublet at zero fields to a fully polarized quartet one brought about by the action of an external magnetic field without Zeeman splitting. This alteration is attributed to the enhancement of the effect of orbital interactions over the spin exchange as the magnitude of the external magnetic field increases.

## 1. Introduction

Since their discovery, lanthanide-based single molecular magnets [1,2,3] have been the subject of great interest to researchers exploring the field of molecular magnetism. From a theoretical perspective, the quest for adequate methods and models to predict the magnetic behavior of these compounds has generated lively debates and a fruitful exchange of ideas in recent years [4,5,6,7,8,9]. By virtue of their intrinsically large magnetic anisotropy, lanthanide series possess a great potential for application in magnetic memory storage nanounits [10,11,12,13] and stand as promising candidates for the realization of quantum logical devices. On the other hand, some lanthanide complexes, such as dysprosium and gadolinium based compounds, are ideal for implementation in magnetic resonance imaging [14,15,16]. Dysprosium complexes play an essential role in gaining useful insights into the magnetic properties of lanthanide-based molecular magnets [17,18,19]. The experimentally observed magnetic bistability at relatively high temperatures in Dy${}^{3+}$ complexes [20,21] and the Dy${}_{5}$ cluster [22], which displays a very high energy barrier of approximately 45.6 meV, are promising candidates for engineering future magnetic molecular devices. Other prominent lanthanide systems are the polyoxometalate-based single molecular magnets, [Ln(W${}_{5}$O${}_{18}$)${}_{2}$]${}^{9-}$, Ln = (Tb, Dy, Ho and Er) [4], with the erbium member (see e.g., Reference [23]) demonstrating a relatively high energy barrier to magnetization reversal.

**Figure 1:**
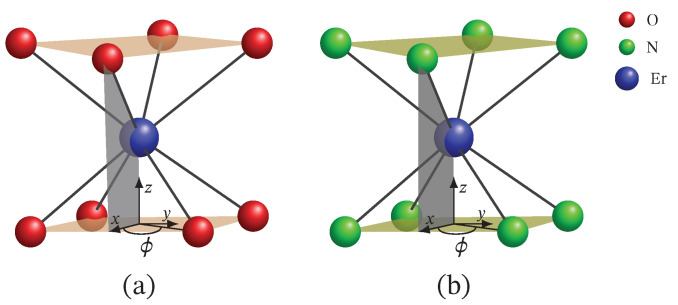
Square antiprismatic structure of Er${}^{3+}$ in: (**a**) Na${}_{9}$[Er(W${}_{5}$O${}_{18}$)${}_{2}$] with twist angle $\phi \approx 42.79^{\circ }$ and structural information reported in Reference [9]; (**b**) [(Pc)Er{Pc{N(C${}_{4}$H${}_{9}$)${}_{2}$}${}_{8}$}]${}^{\cdot }$ with $\phi \approx 43.69^{\circ }$ and structure discussed in Reference [24]. The dihedral angle of the reduced form [(Pc)Er{Pc{N(C${}_{4}$H${}_{9}$)${}_{2}$}${}_{8}$}]${}^{-}$ is $41.40^{\circ }$. The red, green and blue spheres depict oxygen, nitrogen and erbium elements, respectively.

Recently [9], a slow magnetic relaxation in the deuterated species, Na${}_{9}$[Ln(W${}_{5}$O${}_{18}$)${}_{2}$], with Ln = (Tb, Ho and Er) and a field-induced magnetic relaxation in the Ln = Nd member of this group, were reported. For this compound, the Er${}^{3+}$ ion is octacoordinated by four oxygen atoms from each W${}_{5}$O${}_{18}$ group, resulting in a slightly distorted square anti-prismatic geometry, as shown in Figure 1a. The magnetic properties of this material clearly point to a profile characteristic of a single molecular magnet. According to inelastic neutron scattering (INS) measurements, the compound exhibits two ground states and one high-temperature magnetic excitations. The high-temperature transition is characterized by a peculiar high-scattering probability and energy loss of the neutrons. Apart from INS spectra, ac susceptibility measurements performed in the absence of an external magnetic field manifest the compound’s single molecular magnet behavior with an approximately 3.8 meV effective energy barrier. In addition to the slow magnetic relaxation, the dc magnetization and molar susceptibility data suggest a paramagnetic-like behavior. The static magnetic properties are well reproduced by particularly adapted crystal field calculations relying on the electronic structure in Reference [4] and allowing some degree of mixing between some particular states.

Another class of attractive erbium–based single ion magnets includes [(Pc)Er{Pc{N(C${}_{4}$H${}_{9}$)${}_{2}$}${}_{8}$}]${}^{\cdot /-}$ [24]. The Er${}^{3+}$ ion in these compounds is coordinated by eight nitrogen atoms in a square anti-prismatic structure with a different dihedral angle for each compound, see Figure 1b. These compounds possess different field-induced dynamic magnetic properties. In contrast to the reduced compound, [(Pc)Er{Pc{N(C${}_{4}$H${}_{9}$)${}_{2}$}${}_{8}$}]${}^{-}$, which shows slow magnetic relaxation under the action of a dc external magnetic field with an energy barrier of approximately 0.73 meV, the unprotonated compound, [(Pc)Er{Pc{N(C${}_{4}$H${}_{9}$)${}_{2}$}${}_{8}$}]${}^{\cdot }$, shows no trace of a relaxation pathway above 2 K and for a dc field B=0.1 T. In the absence of a static magnetic field, both compounds are indistinguishable with respect to ac susceptibility data. On the other hand, they demonstrate similar dc magnetic behavior with a small difference with regard to the saturation of the magnetization. The reduced compound exhibits a lower saturation value of the magnetization and, in contrast to that of the neutral compound, it is almost temperature-independent within the range 2–6 K. It is worth noting that comparisons between theoretical results and experimental data for the overall magnetic properties are not reported. Furthermore, to our knowledge, experimental data for the magnetic spectrum of both compounds have not been reported so far.

In this paper, we study to what extent an effective spin exchange Hamiltonian may rationalize the magnetic properties of Er${}^{3+}$ single ion magnets. To this end, we adapt a recently proposed spin–sigma model [25] and focus on the erbium-based molecular magnets, Na${}_{9}$[Er(W${}_{5}$O${}_{18}$)${}_{2}$] and [(Pc)Er{Pc{N(C${}_{4}$H${}_{9}$)${}_{2}$}${}_{8}$}]${}^{\cdot /-}$. The proposed model is developed within the multi-configurational self-consistent field method, and was successfully used to characterize the magnetism of the molecular magnet, Ni${}_{4}$Mo${}_{12}$ [26,27], and the magnetic spectrum of the trimeric compounds, A${}_{3}$Cu${}_{3}$(PO${}_{4}$)${}_{4}$ A=(Ca, Sr, Pb) [28]. This formalism describes all electrons as delocalized occupying molecular orbitals. Therefore, we show that, besides the use of localized electron states in the free-ion approximation and the crystal field formalism, there may exist an appropriate complete active space and spin coupling scheme, where the named model is able to reproduce the respective experimental findings reported in the literature [9,24]. In addition to the spin–sigma model, we apply the Heisenberg one and demonstrate that it fails at providing us with a complete picture of the corresponding magnetic properties.

For the complex Na${}_{9}$[Er(W${}_{5}$O${}_{18}$)${}_{2}$], we compute in detail the inelastic neutron scattering intensities and hence deduce the minimal effective energy levels sequence describing the experimentally observed magnetic excitations. Furthermore, we obtain theoretical results consistent with the magnetization and susceptibility measurements, providing us with the overall field-dependent energy spectrum in both the dc and ac regimes. We demonstrate that the effective energy barrier to magnetization reversal satisfies the inequality $3.81\;\mathrm{meV}\leq U_{\mathrm{eff}}\leq 3.94$ meV, which agrees very well with the experimental value of 3.8 meV [9], explaining the slow magnetic relaxation. The model further suggests an abrupt change in the ground state from a non-fully polarized spin ground state in the absence of an external magnetic field to a fully spin polarized one that does not result from the decreased spin rotational degeneracy caused by the relevant Zeeman interactions, but rather orbital ones that affect the system’s energy indirectly due to the used multi-configurational basis, see Reference [25].

The magnetic properties of single ion magnets, [(Pc)Er{Pc{N(C${}_{4}$H${}_{9}$)${}_{2}$}${}_{8}$}]${}^{\cdot /-}$, are also computed. Our results are quantitatively and qualitatively in good agreement with the existing magnetization, dc and ac susceptibility experimental data. The calculated value of the effective energy barrier to magnetization reversal for the reduced compound is consistent with the experimentally obtained one [24]. With respect to the static magnetic properties, the spin–sigma model predicts a low-field induced change in the ground state from a non-fully to a fully polarized spin state, driven by the indirect external field effect rather than the direct spin Zeeman interactions. Further, calculating the temperature dependence of the magnetization, we found a small variation in the *g*-factor value in the case of the reduced form [(Pc)Er{Pc{N(C${}_{4}$H${}_{9}$)${}_{2}$}${}_{8}$}]${}^{-}$. According to the applied method, such changes may be attributed to some nuclei spins that are uncoupled due to thermal effects or a change in the associated *g*-factor anisotropy. Experimental data for the magnetic spectrum of both compounds are unavailable in the literature and all spectroscopic model parameters are fitted only to the available magnetization and susceptibility measurements.

The rest of the paper is organized as follows: In Section 2, we present the physical models used to explore the magnetic properties of the considered compounds and introduce the relevant parameters, the used approximations and the pertinent physical relations. Our results for the magnetic spectrum, magnetization and susceptibility of the compound Na${}_{9}$[Er(W${}_{5}$O${}_{18}$)${}_{2}$], along with the corresponding analysis, are reported in Section 3. In Section 4, we compute and discuss the results for the magnetic properties of bis(phthalocyaninato) double-decker compounds [(Pc)Er{Pc{N(C${}_{4}$H${}_{9}$)${}_{2}$}${}_{8}$}]${}^{\cdot /-}$. Section 5 summarizes the results.

## 2. The Hamiltonian

To study the experimentally observed magnetic properties of the compounds Na${}_{9}$[Er(W${}_{5}$O${}_{18}$)${}_{2}$] [9] and [(Pc)Er{Pc{N(C${}_{4}$H${}_{9}$)${}_{2}$}${}_{8}$}]${}^{\cdot /-}$ [24], we rely on the method proposed in Reference [25]. Here, we assume that a single molecule hosts no more than three unpaired valence electrons in its ground state with all non-bonding orbitals being fully occupied. The three active molecular orbitals are antibonding, such that the first one has lower energy than that of the 2-nd and 3-rd orbitals. Further, the quantization axis is oriented along the *z* direction and the effective magnetic centers associated to the three electrons and active molecular orbitals are characterized by the same *g*-factors value. Within the considered molecular orbital structure, we have three effective magnetic centers with a spin quantum number, $s_{i}=\frac{1}{2}$, i=1,2,3. Therefore, we have the spin coupling scheme $\left\{s_{23}^{},s\right\}$, with $|s_{2}^{}-s_{3}^{}|\leq s_{23}^{}\leq \left|s_{2}^{}+s_{3}^{}\right|$ and $|s_{23}^{}-s_{1}^{}\left|\leq s\leq \right|s_{23}^{}+s_{1}|$, where $s_{23}$ indicates the singlet $s_{23}=0$ and triplet $s_{23}=1$ spin–orbital configurations related to the 2-nd and the 3-rd molecular orbitals, *s* is the effective total spin quantum number of the resulting Er${}^{3+}$ magnetic center.

Under the considered assumptions and the introduced effective spin coupling scheme, the spin–sigma Hamiltonian reads (see e.g., Reference [25])

(1) $$\hat{H}=J\left({\hat{\sigma }}_{23}^{}\cdot {\hat{s}}_{1}^{}+{\hat{\sigma }}_{1}^{}\cdot {\hat{s}}_{23}^{}+{\hat{\sigma }}_{2}^{}\cdot {\hat{s}}_{3}^{}+{\hat{\sigma }}_{3}^{}\cdot {\hat{s}}_{2}^{}\right)-\mu_{B}B\sum_{i,\alpha }{\hat{S}}_{i}^{\alpha },$$

where J is the intramolecular exchange parameter between the spin centers, the effective three components’ spin operator ${\hat{s}}_{i}$ and the spin-like operator $\hat{\sigma_{i}}$ are associated to the *i*-th magnetic center. The operator ${\hat{S}}_{i}^{\alpha }$ in the field dependent term satisfies the relation

$${\hat{S}}_{i}^{\alpha }{|s_{23},s,m\rangle }_{n_{s_{23},s}}=g_{\alpha }{\hat{s}}_{i}^{\alpha }{|s_{23},s,m\rangle }_{n_{s_{23},s}},\;\;\forall \;i,\alpha ,$$

where ${\hat{s}}_{i}^{\alpha }$, with α∈{x,y,z}, is the α-th component of the *i*-th spin operator and

$$B=\sqrt{B^{2}+Bb\cos\left(\omega t\right)+b^{2}}.$$

Here, *B* is the magnitude of the dc magnetic field and *b* is that of the alternating field with frequency ω. Under the action of an external magnetic field, the operators $\hat{\sigma_{i}}$ obey the relations

$$\begin{array}{cc} \; & {\hat{\sigma }}_{1}^{}{|s_{23}^{},s,m\rangle }_{n_{s_{23},s}}=h_{s}{\hat{s}}_{1}^{}{|s_{23}^{},s,m\rangle }_{n_{s_{23},s}},\\ \; & {\hat{\sigma }}_{i}^{}{|s_{23}^{},s,m\rangle }_{n_{s_{23},s}}=h_{s}c_{23,n_{s_{23},s}}^{s_{23},s}{\hat{s}}_{i}^{}{|s_{23}^{},s,m\rangle }_{n_{s_{23},s}},\;\;i=2,3. \end{array}$$

Notice that, for all $s_{23}^{}$, *s* and *m*, $g_{\alpha }$ is the α-th component of the effective *g*-factor, say g, $c_{23,n_{s_{23},s}}^{s_{23},s}$ and $h_{s}$ are the corresponding spectroscopic and field parameters, respectively. Moreover, we would like to point out that the model parameters, related to Hamiltonian (1), account for the contribution of all electrons occupying core molecular orbitals and that, due to the lack of exchange bridges, the value of the *g*-factor does not depend directly on any of the three good spin quantum numbers as the general case described in Reference [25] suggests. For more details about the physics behind all model parameters, the reader may consult Reference [25].

The eigenvalues of (1) are given by

(2) $$\begin{array}{cc} E_{s_{23}^{},s,m}^{f,(n_{s_{23}^{},s})}=\;\; & \frac{Jh_{s}}{2}\left(1+c_{23,n_{s_{23},s}}^{s_{23},s}\right)\left[s\left(s+1\right)-s_{23}^{}\left(s_{23}^{}+1\right)-\frac{3}{4}\right]\\ \; & +Jh_{s}c_{23,n_{s_{23},s}}^{s_{23},s}\left[s_{23}^{}\left(s_{23}^{}+1\right)-\frac{3}{2}\right]-mg\mu_{B}B, \end{array}$$

where *n*${}_{1,1/2}$=1, *n*${}_{1,3/2}$=1, *n*${}_{0,1/2}$=1,2 and g=|g|.

For a thorough study of the dc magnetic properties of the considered compounds and for the sake of comparison, in addition to the spin–sigma Hamiltonian given in (1), we compute the same properties in the framework of the Heisenberg model. For B=B, the corresponding Hamiltonian reads

(3) $$\hat{H}=2J{\hat{s}}_{23}^{}\cdot {\hat{s}}_{1}^{}+2J_{23}^{}{\hat{s}}_{2}^{}\cdot {\hat{s}}_{3}^{}-\mu_{B}B\sum_{i,\alpha }{\bar{g}}_{\alpha }{\hat{s}}_{i}^{\alpha },$$

where *J* and $J_{23}$ are the corresponding exchange parameters, ${\bar{g}}_{\alpha }=g_{e}n_{\alpha }$ is the α-th component of the corresponding *g*-factor with $g_{e}$ denoting the electron’s *g*-factor and $n_{\alpha }$ being the α-th component of the unit vector n that defines the direction of the externally applied magnetic field.

The eigenvalues of (3) are

(4) $$E_{s_{23}^{},s,m}=J\left[s\left(s+1\right)-s_{23}^{}\left(s_{23}^{}+1\right)-\frac{3}{4}\right]+J_{23}\left[s_{23}^{}\left(s_{23}^{}+1\right)-\frac{3}{2}\right]-mg_{e}\mu_{B}B,$$

where $|s_{23}^{},s\rangle$ are the respective eigenstates in the absence of the external magnetic field, that is, B=0.

The difference between $g_{\alpha }$ and ${\bar{g}}_{\alpha }$ for all α components is that, in contrast to (1), the Zeeman term in (3) does not account for the magnetic field induced by all remaining electrons in the molecule. Thus, for a single electron system $g_{\alpha }\equiv {\bar{g}}_{\alpha }$, see Reference [25]. It is worth mentioning that, since for rare earth elements the spin–orbital coupling is not a perturbation to the crystal field effect, the inclusion of the g-tensor is irrelevant.

We would like to emphasize that the energy spectra in (2) and (4) map only those energy levels from the initial variational spectrum that are relevant to the magnetic properties of the considered systems. Therefore, even the low-lying excited states related to transitions between molecular orbitals of different energies are ruled out.

## 3. The Single Ion Magnet Na${}_{9}$[Er(W${}_{5}$O${}_{18}$)${}_{2}$]

### 3.1. Energy Spectrum

**Figure 2:**
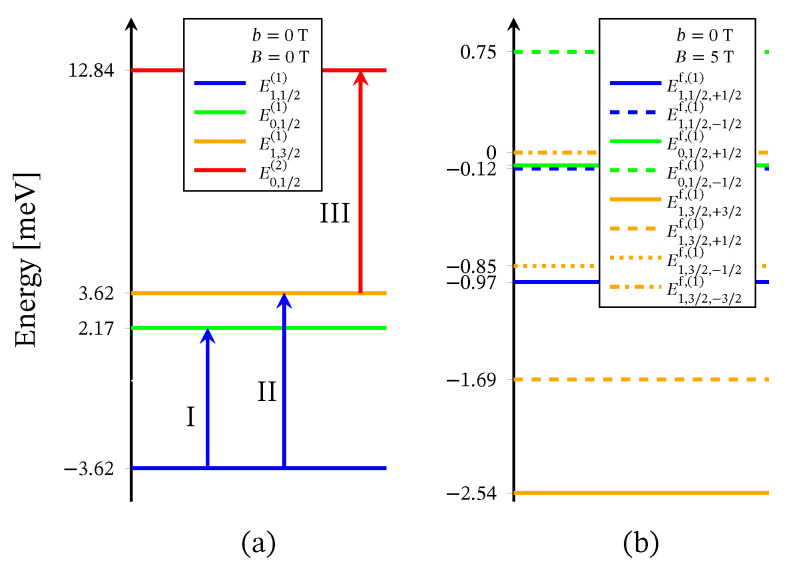
Effective energy spectrum of the polyanion [Er(W${}_{5}$O${}_{18}$)${}_{2}$]${}^{9-}$ obtained with the aid of Hamiltonian (1), (**a**) in the absence of external magnetic field and (**b**) with applied field. The blue and red arrows show the ground state and the high-temperature magnetic excitation energies, respectively, see Figure 3.

**Figure 3:**
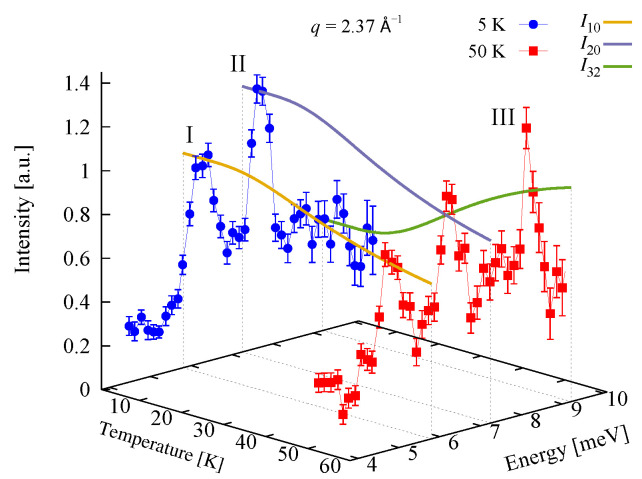
Experimental and calculated INS intensities of the single ion magnet Na${}_{9}$[Er(W${}_{5}$O${}_{18}$)${}_{2}$] as a function of neutrons’ energy transfer and temperature. Experimental data are taken from Reference [9]. The solid lines depict the calculated intensities $I_{n^{\prime }n}$, where $n^{\prime }$ and *n* denote the final and initial energy levels of the transitions shown in Figure 2a. The curves for $I_{10}$ and $I_{20}$ obtained from the spin–sigma and Heisenberg Hamiltonians overlap.

**Figure 4:**
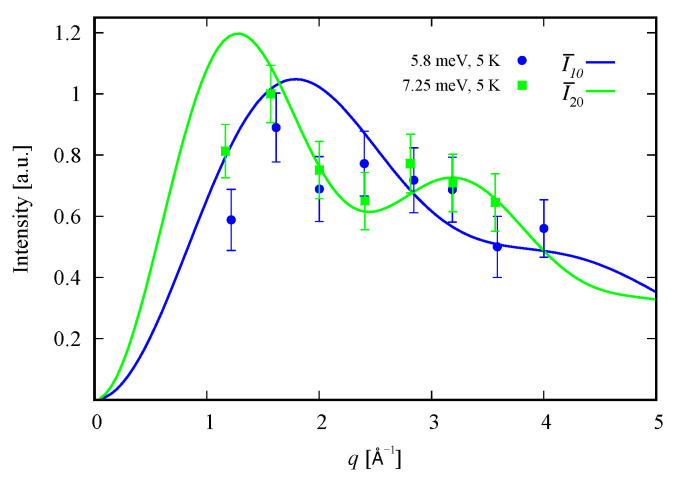
Normalized by $\gamma_{n^{\prime }n}$ INS intensities (solid lines) as a function of the magnitude of the scattering vector *q* along with the experimental data of Reference [9] for Na${}_{9}$[Er(W${}_{5}$O${}_{18}$)${}_{2}$]. Here, ${\bar{I}}_{n^{\prime }n}=I_{n^{\prime }n}/\gamma_{n^{\prime }n}$, where $n^{\prime }$ and *n* denote the final and initial energy levels of the transitions shown in Figure 2a. The curves for ${\bar{I}}_{10}$ and ${\bar{I}}_{20}$ obtained with the aid of spin–sigma and Heisenberg Hamiltonians overlap.

**Figure 5:**
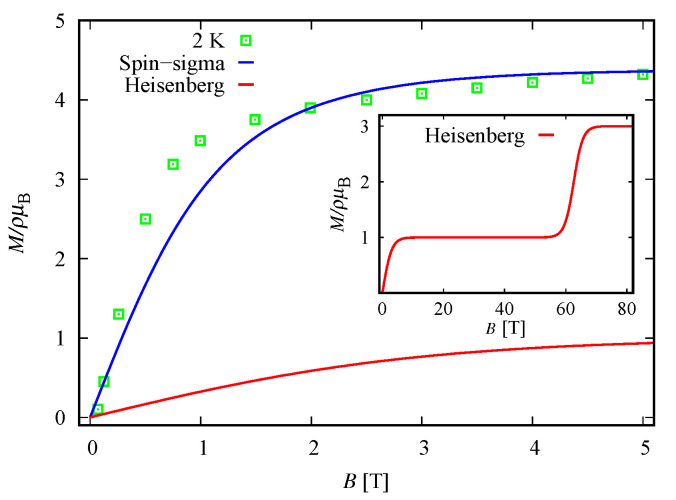
Non-dimensional magnetization of the complex Na${}_{9}$[Er(W${}_{5}$O${}_{18}$)${}_{2}$] as a function of dc magnetic field at 2 K and b=0 T. Here, ρ and $\mu_{B}$ denote the number of isolated polyanions per unit volume and Bohr magneton, respectively. The experimental data, the green squares, are provided in Reference [9]. The solid blue and red lines represent the calculated magnetization obtained, respectively, by using Hamiltonian (1) and the Heisenberg model (3). The inset shows a saturation of magnetization with values above 70 T predicted from the Heisenberg model.

**Figure 6:**
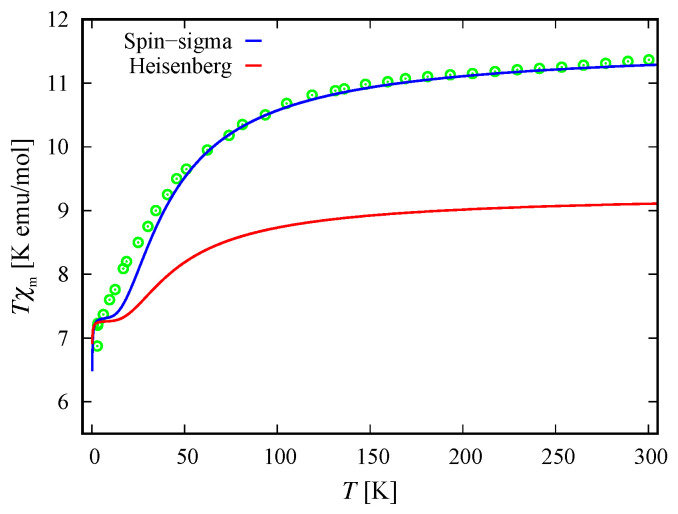
Molar susceptibility of Na${}_{9}$[Er(W${}_{5}$O${}_{18}$)${}_{2}$] as a function of temperature, multiplied by the temperature. The experimental data (green circles) from Reference [9] are shown along with the theoretical results (solid lines) for B=0.1 T and b=0 T. The susceptibility obtained with the aid of spin–sigma Hamiltonian (1) is depicted by a blue line. The red one shows the calculations performed with the Heisenberg model.

The energy levels sequence obtained from (2) is shown in Figure 2. For B=0 the energy spectrum consists of three doublet and one quartet levels. The presence of three doublet energy levels is traced back to the existence of distinct by energy two singlet configurations associated to the 2-nd and the 3-rd molecular orbitals. In particular, the third doublet, with energy *E*${}_{0,1/2}^{\left(2\right)}$, is associated to the local singlet $s_{23}=0$ in which two of the three electrons occupy the same molecular orbital. The corresponding doublet energies, *E*${}_{0,1/2,\pm 1/2}^{f,(2)}$, are not included in Figure 2b, due to the zero probability of observing the respective states in the case B≠0 and in the absence of perturbation interactions involving the neutrons. The spectroscopic parameters ‘*c*’ are fixed with respect to the inelastic neutron scattering experiments depicted on Figure 3 and Figure 4. The values of the ‘*h*’-field parameters are determined at the saturation of the magnetization at B>3 T and b=0 T, see Figure 5 and Figure 6. The corresponding values are given in Table 1.

**Figure 7:**
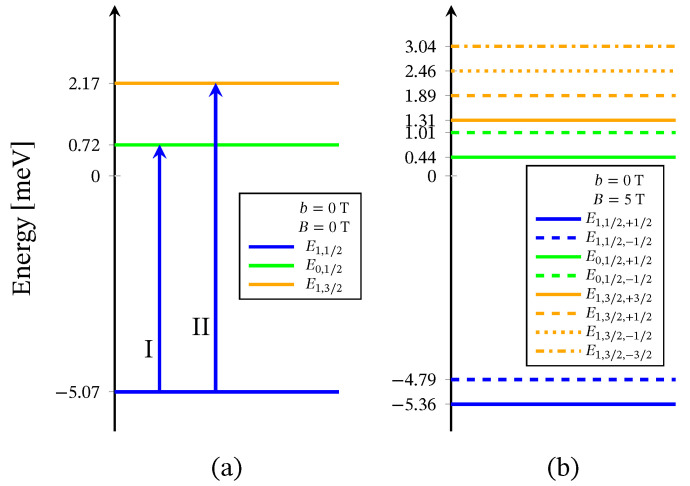
Heisenberg energy spectrum for the polyanion [Er(W${}_{5}$O${}_{18}$)${}_{2}$]${}^{9-}$, (**a**) in the absence of external magnetic field, (**b**) with applied field. The blue arrows show the two ground state magnetic excitations, see Figure 3.

The Heisenberg energy spectrum (4) is constructed of two doublet and one quartet energy levels, see Figure 7. The values of both exchange parameters are determined via the inelastic neutron scattering data shown on Figure 3 and Figure 4.

### 3.2. Magnetic Spectrum

INS measurements for the compound Na${}_{9}$[Er(W${}_{5}$O${}_{18}$)${}_{2}$], reported in Reference [9], show the magnetic spectrum exhibiting two low-temperature and one high-temperature peaks. The energy of the three magnetic excitations depicted by Roman numbers on Figure 3 are approximately

(5) $$\Delta_{I}=5.8\;\mathrm{meV},\;\Delta_{\mathrm{II}}=7.25\;\mathrm{meV},\;\Delta_{\mathrm{III}}=9.22\;\mathrm{meV}.$$

All three magnetic peaks are characterized by the spin–sigma energy spectrum (2), with parameters

$$J=\frac{1}{3}\Delta_{\mathrm{II}},\;c_{23,1}^{0,1/2}=1-2\frac{\Delta_{I}}{\Delta_{\mathrm{II}}},\;c_{23,2}^{0,1/2}=1+2\frac{\Delta_{\mathrm{III}}}{\Delta_{\mathrm{II}}}.$$

The corresponding transitions are depicted in Figure 2a by arrows. The parameters’ values are given in Table 1.

The energy spectrum (4) detects only the magnetic peaks I and II, with exchange parameters

$$J=\frac{1}{3}\Delta_{\mathrm{II}},\;J_{23}=\frac{1}{3}\Delta_{\mathrm{II}}-\frac{1}{2}\Delta_{I},$$

where, according to (5), we have J=2.41(6) meV and $J_{23}=-0.48\left(3\right)$ meV. Both ground state transitions are shown in Figure 7 by blue arrows. The energy spectrum (4) cannot reproduce the high-temperature transition designated as III, since for $s_{23}=0$ and s=1/2, the Heisenberg model is unable to account for the probability of observing two distinct by energy singlet spin-orbital configurations. Thus, working with the Heisenberg model, one can take into consideration only one transition with regard to a particular configuration state function [29].

### 3.3. Inelastic Neutron Scattering Intensities

INS intensities for each of the three magnetic peaks shown by Roman numbers in Figure 3 are computed. According to the considered effective spin coupling scheme, we have the selection rules $\Delta s_{23}=0,\pm 1$, Δs=0,±1 and Δm=0,±1. The analytical results along with the experimental data [9] for the temperature *T* dependence and that of the magnitude of the scattering vector *q* are depicted in Figure 3 and Figure 4, respectively.

For the integrated intensity of the first magnetic excitation with energy $\Delta_{I}$, we get

(6) $$I_{10}\left(q,T\right)\propto \frac{4}{3}p_{0}\left(T\right)F^{2}\left(q\right)\left[1-\frac{\sin(qr)}{qr}\right],$$

where r=2.315 Å is the distance between the effective spin–half magnetic centers associated to the electrons occupying the 2-nd and the 3-rd molecular orbitals, $p_{0}\left(T\right)$ is the probability distribution related to the ground state. The from factor is obtained with respect to the 4f subshell atomic states of the erbium atom. It reads

$$F\left(q\right)=\frac{z^{10}\left(0.406z^{6}-3.178q^{2}z^{4}+3.557q^{4}z^{2}-0.568q^{6}\right)}{{\left(q^{2}+0.8934z^{2}\right)}^{8}},$$

where the effective charge is given by z=27.978 [30].

For the INS integrated intensity of the second peak with neutrons’ energy loss equal to $\Delta_{\mathrm{II}}$ we have

(7) $$I_{20}\left(q,T\right)\propto \frac{24}{9}p_{0}\left(T\right)F^{2}\left(q\right)\left[1+\frac{\sin(qr)}{3qr}-\frac{4\sin(qr^{\prime })}{3qr^{\prime }}\right],$$

where $r^{\prime }=3.194$ Å is the distance between the 1-st effective magnetic center and the remaining two that correspond to the electrons residing in the 2-nd and the 3-rd orbitals.

The high-temperature magnetic excitation can be reproduced only with the aid of the spin–sigma model (1). The intensity is

(8) $$I_{32}\left(q,T\right)\propto 8p_{2}\left(T\right)F^{2}\left(q\right)\left[1+\frac{3\cos(qr)}{2q^{2}r^{2}}-\left(\frac{3}{2}+\frac{11q^{2}r^{2}}{12}\right)\frac{\sin(qr)}{q^{3}r^{3}}\right],$$

where the probability $p_{2}\left(T\right)$ is associated to the second excited state with energy $E_{1,3/2}^{\left(1\right)}=3.62$ meV shown in Figure 2a. In computing the intensity (8), we accounted for all sets of non-magnetic states preserving the quantum number $s=\frac{3}{2}$, including the spin–quadrupole ones.

The normalization factors used to depict the *q* dependence on Figure 4 are given by

(9) $$\gamma_{n0}\left(T\right)=2n\frac{(n+1)!}{3^{n}}p_{0}\left(T\right),\;n=1,2.$$

### 3.4. Magnetization and Susceptibility

In order to understand the magnetic behavior of the compound Na${}_{9}$[Er(W${}_{5}$O${}_{18}$)${}_{2}$], in addition to the analysis of the magnetic spectrum, we explore the magnetization and the susceptibility behavior. According to the Zeeman terms in (1) and (3), the magnetization reads M=Mn, with

(10) $$M=\kappa T\vartheta^{-1}\partial_{B}\ln Z,\;Z\left(T,B\right)=\mathrm{Tr}\;e^{-\hat{\Sigma }\left(B\right)/\kappa T},$$

where κ is the Boltzmann constant, ϑ is a unit volume and $\hat{\Sigma }\in \left\{\hat{H},\hat{H}\right\}$ is the Hamiltonian of the system. The theoretical results for the corresponding dc non-dimensional magnetization of the isolated polyanion are depicted in Figure 5 along with the experimental data from Reference [9], where ρ=N/ϑ with *N* indicating the number of isolated complexes.

The values of the ‘*h*’-field parameters are determined with respect to the magnetization data, see Table 1. Introduced as a function of the energy gaps of the relevant spectrum, leading to

$$\begin{array}{cc} \; & h_{1/2}=\frac{E_{0,1/2,m}^{f,(1)}-E_{1,1/2,m}^{f,(1)}}{\Delta_{I}},\\ \; & h_{3/2}=\frac{E_{1,3/2,m}^{f,(1)}-E_{1,1/2,m}^{f,(1)}}{E_{1,3/2}^{\left(1\right)}}-\frac{E_{0,1/2,m}^{f,(1)}-E_{1,1/2,m}^{f,(1)}}{\Delta_{I}}, \end{array}$$

where m=±1/2.

With respect to (10) the molar susceptibility reads

(11) $$\chi_{m}^{}=\mu \rho^{-1}\chi ,\;\chi =\mu_{o}\partial_{B}M,$$

where μ is the molar mass and $\mu_{o}$ is the vacuum permeability. For $\mu \rho^{-1}=2.055$${\mathrm{mol}}^{-1}$ and B=B=0.1 T, the dc molar susceptibility multiplied by *T* is shown in Figure 6. It suggests a non-fully polarized spin ground state in a low-field regime, which is in agreement with the obtained ground state in the case B=0 for both the spin–sigma and Heisenberg Hamiltonians. However, the magnetization depicted in Figure 5 rapidly increased against the magnitudes of the external magnetic field and quickly reaches saturation values, signaling a possible ground state of fully polarized spins even at low-field values. In the framework of the considered method, and hence the spin–sigma Hamiltonian, such behavior may be interpreted as an abrupt change in the ground state from a doublet at B=0 to a quartet for B≠0 due to orbital contributions. In other words, when a magnetic field is applied, the contribution of electrons’ orbital moments into the energy of the different spin–orbital configurations increases, thus changing the relevant energy levels sequence, see Reference [25]. This effect is accounted for by the ‘*h*’-field parameters. In this respect, the Heisenberg model fails to reproduce the magnetization data and predicts a very broad intermediate step shown in the inset in Figure 5.

### 3.5. Susceptibility in ac Field

**Figure 8:**
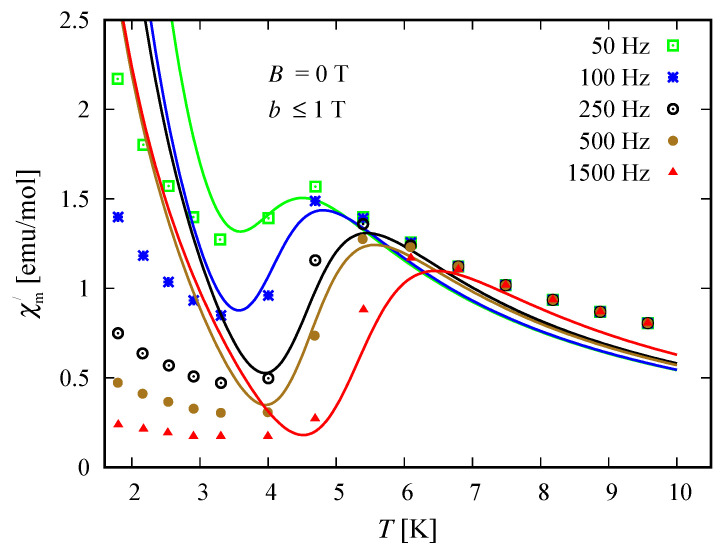
Temperature dependence of the in-phase susceptibility for the compound Na${}_{9}$[Er(W${}_{5}$O${}_{18}$)${}_{2}$]. The symbols depict the experimental data from Reference [9]. The theoretical results obtained with the aid of the spin–sigma model (1) and (12), at an amplitude of the ac field b≤1, are presented with solid lines.

**Figure 9:**
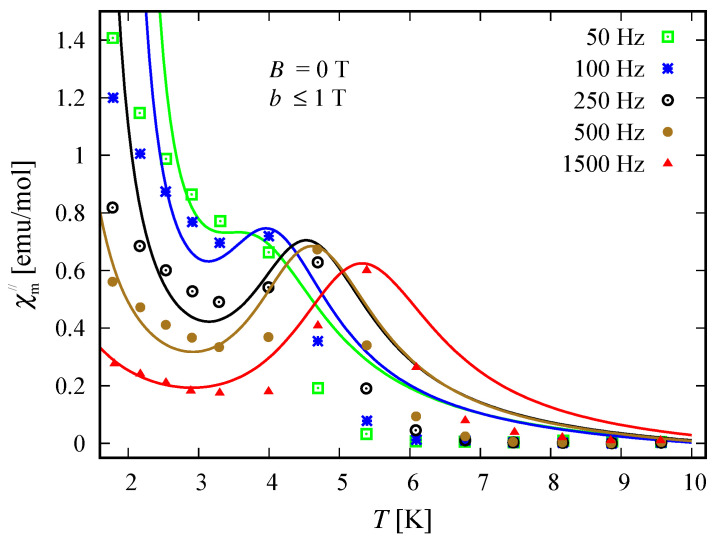
Out-of-phase susceptibility of the compound Na${}_{9}$[Er(W${}_{5}$O${}_{18}$)${}_{2}$] as a function of the temperature. The experimental and theoretical results are shown by symbols and solid lines, respectively. Experimental data are taken from Reference [9]. Calculations are performed using (12) and the spin–sigma model (1), with the magnitude of the ac field b≤1 T.

The compound Na${}_{9}$[Er(W${}_{5}$O${}_{18}$)${}_{2}$] exhibits a single molecular magnet behavior in the absence of a static magnetic field, which is nicely described by the spin–sigma model. A comparison between the results obtained with the aid of (2) and the experimental data from Reference [9] is depicted in Figure 8 and Figure 9.

From (11), we calculate the in-phase susceptibility using the relation

(12a) $$\chi_{m}^{\prime }=c_{1}\frac{\chi_{m}^{}}{1-d_{1}\chi }+u_{1}\frac{a_{1}\chi_{m}^{}-\mu \rho^{-1}\left(a_{1}^{2}+a_{2}^{2}\right)\chi^{2}}{{\left(1-a_{1}\chi \right)}^{2}+a_{2}^{2}\chi^{2}},$$

and for the out-of-phase susceptibility,

(12b) $$\chi_{m}^{\prime \prime }=c_{2}\frac{\chi_{m}^{}}{1-d_{2}\chi }+u_{2}\frac{a_{2}\chi_{m}^{}}{{\left(1-a_{1}\chi \right)}^{2}+a_{2}^{2}\chi^{2}},$$

where the real scalars $c_{1}$, $c_{2}$, $u_{1}$ and $u_{2}$ satisfy the conditions $c_{1}=1$, $c_{2}=0$, $u_{1}=0$ and $u_{2}=0$ at ω=0. Furthermore, $d_{1}\equiv d_{1}\left(\omega ,\tau \right)$, $d_{2}\equiv d_{2}\left(\omega ,\tau \right)$, $a_{1}\equiv a_{1}\left(\omega ,\tau \right)$ and $a_{2}\equiv a_{2}\left(\omega ,\tau \right)$ are real functions, such that $d_{1}\left(0,\tau \right)=d$, $d_{2}\left(0,\tau \right)=d$, $a_{1}\left(0,\tau \right)=1$ and $a_{2}\left(0,\tau \right)=0$, with *d* denoting the demagnetization factor and τ the relaxation time. All parameters have finite values in the high-frequency limit. For example, we have $\lim_{\omega \to \infty }a_{1}\left(\omega ,\tau \right)\to 0$ and $\lim_{\omega \to \infty }a_{2}\left(\omega ,\tau \right)\to 0$. Moreover, in the case of negligible energy difference to the relaxation pathway, we have $c_{2}=0$, $a_{1}\left(\omega ,\tau_{0}\right)=0$ and $a_{2}\left(\omega ,\tau_{0}\right)=0$, where $\tau_{0}$ denotes the corresponding trial time.

All fitting parameters are listed in Table 2. The values of the ‘*h*’-field parameters are significantly reduced indicating a relatively large energy barrier leading to a slow magnetic relaxation. Accordingly, the model predicts an energy barrier with a value in the interval $3.81\;\mathrm{meV}\leq U_{\mathrm{eff}}\leq 3.94$ meV, which is compatible with the experimental findings of approximately 3.8 meV [9]. As in the case of static magnetic properties, this result points to a significant contribution of the electrons’ orbitals. Nevertheless, the spin–sigma model is unable to provide details about the relevant relaxation pathway. To resolve this issue, one has to work with the underlying variational energy spectrum [25]. This, however is very demanding and requires huge computational efforts.

## 4. The Single ion Magnets [(Pc)Er{Pc{N(C${}_{4}$H${}_{9}$)${}_{2}$}${}_{8}$}]${}^{\cdot /-}$

### 4.1. Energy Spectra

**Figure 10:**
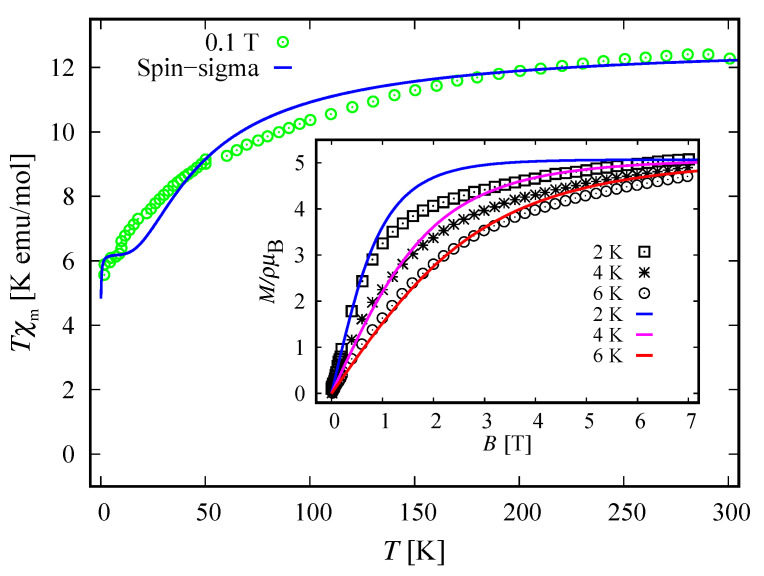
Experimental data from Reference [24] and calculated molar susceptibility of the bis(phthalocyaninato) Er${}^{3+}$ complex [(Pc)Er{Pc{N(C${}_{4}$H${}_{9}$)${}_{2}$}${}_{8}$}]${}^{\cdot }$ as a function of temperature, multiplied by the temperature, for B=0.1 T and b=0 T. The inset shows the dc field dependence of the corresponding non-dimensional magnetization in the case of three different temperatures, where ρ and $\mu_{B}$ are the number of isolated complexes per unit volume and Bohr magneton, respectively. In both figures, the solid lines represent the theoretical results obtained with respect to the spin–sigma Hamiltonian (1).

**Figure 11:**
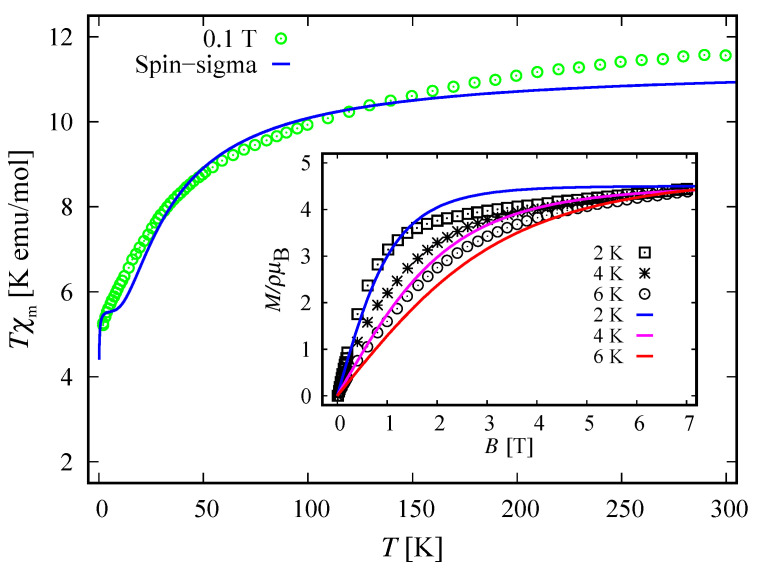
Molar susceptibility of the compound [(Pc)Er{Pc{N(C${}_{4}$H${}_{9}$)${}_{2}$}${}_{8}$}]${}^{-}$ as a function of temperature, multiplied by the temperature at B=0.1 T and b=0 T. The inset depicts the corresponding non-dimensional magnetization as a function of the dc field for three different temperatures, with ρ and $\mu_{B}$ designating the number of isolated complexes per unit volume and Bohr magneton, respectively. In both figures, the solid lines represent the theoretical results obtained with respect to the spin–sigma Hamiltonian (1). The circles, squares and stars represent the experimental data from Reference [24].

**Figure 12:**
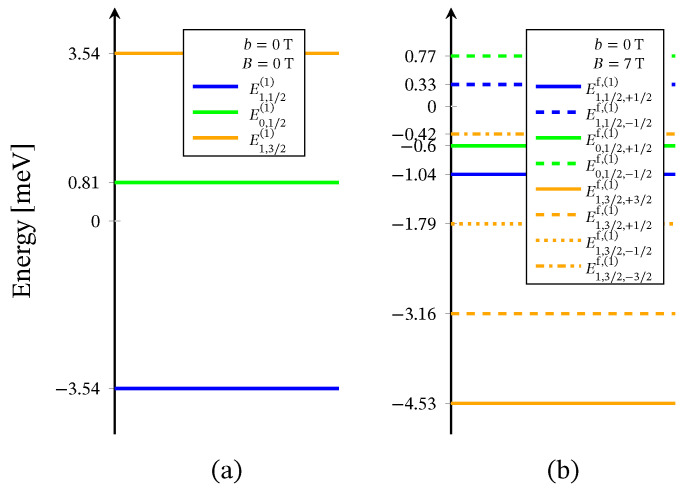
Effective energy spectrum of the compound [(Pc)Er{Pc{N(C${}_{4}$H${}_{9}$)${}_{2}$}${}_{8}$}]${}^{\cdot }$ obtained with Hamiltonian (1) only with respect to the magnetization and susceptibility data from Reference [24], shown in Figure 10. Here, (**a**) depicts the predicted energy levels sequence for the case B=0 T and (**b**) with applied field of 7 T.

**Figure 13:**
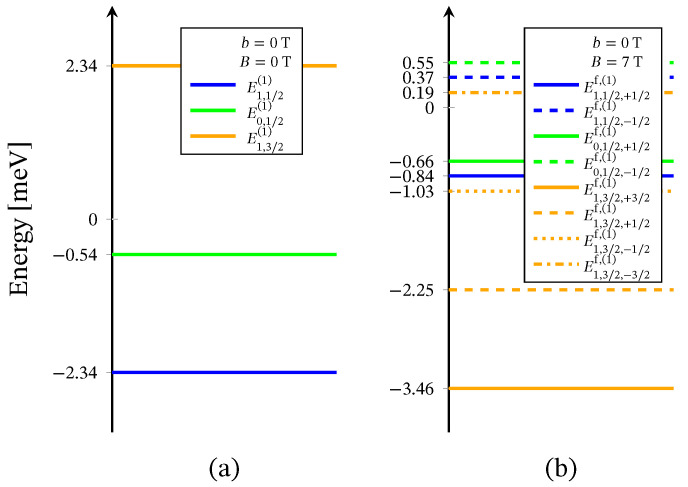
Effective energy spectrum of the compound [(Pc)Er{Pc{N(C${}_{4}$H${}_{9}$)${}_{2}$}${}_{8}$}]${}^{-}$ calculated with the aid of (1). The parameters are fitted according to the magnetization and susceptibility data from Reference [24], depicted on Figure 11. Subfigure (**a**) shows the energy levels sequence for B=0 T and (**b**) in the case of applied field of 7 T, for g=3.

Similar to the case of the polyanion [Er(W${}_{5}$O${}_{18}$)${}_{2}$]${}^{9-}$, Hamiltonian (3) is not able to describe the magnetic properties of the compounds [(Pc)Er{Pc{N(C${}_{4}$H${}_{9}$)${}_{2}$}${}_{8}$}]${}^{\cdot /-}$. In this respect, we use only Hamiltonian (1), whose eigenvalues are given by (2). Since no measurements for these compounds’ magnetic spectra are reported, all model parameters are fitted to the dc magnetization and the susceptibility data provided in Reference [24] and depicted in Figure 10 and Figure 11. The energy spectra of the neutral and the reduced compounds are shown in Figure 12 and Figure 13, respectively. For both materials, in the absence of an external magnetic field, we have a doublet ground state energy level corresponding to the local triplet $s_{23}=1$ and a doublet level with $s_{23}=0$ related to the first excited state. The second excited level is related to the quartet states. Although in the case B≠0, the magnetization and susceptibility data may shed light on the sequence of the first two excited energy levels, the existence of a third excited level that is allowed by the spin–sigma model remains questionable. Similar to the effect observed in [Er(W${}_{5}$O${}_{18}$)${}_{2}$]${}^{9-}$ and shown in Figure 2b, in the presence of the dc magnetic field, the used model predicts a faster than expected change in the ground state from a doublet to a fully polarized quartet approaching saturation of magnetization, see Figure 12b and Figure 13b.

### 4.2. Magnetization and Susceptibility

The respective dc magnetization and molar susceptibility are calculated using (10) and (11) with B=B. For both compounds we obtain $\mu \rho^{-1}=2.38$${\mathrm{mol}}^{-1}$. The comparison between theory and experiment is depicted in Figure 10 and Figure 11. The fully polarized spin state suggested by the magnetization data versus the non-fully polarized one demonstrated by the behavior of low-field susceptibility as a function of temperature is explained by the rapid change in the ground state due to increased orbital contributions for B≠0, see Reference [25]. In general, both compounds demonstrate the same magnetic behavior. Nevertheless, there is a feature that can be used to distinguish between both molecules. The saturation of magnetization for the unprotonated compound [(Pc)Er{Pc{N(C${}_{4}$H${}_{9}$)${}_{2}$}${}_{8}$}]${}^{\cdot }$ slowly decreases with increasing temperature, which is plausible from a theoretical point of view, see the inset of Figure 10. In contrast, the saturation value of the magnetization for [(Pc)Er{Pc{N(C${}_{4}$H${}_{9}$)${}_{2}$}${}_{8}$}]${}^{-}$, depicted on the inset of Figure 11, shows no signs of temperature dependence in the range 2–6 K. As a result, we observe a small change in the total *g*-factor value, from g=3 in the domain 2–4 K to g=3.15 at 6 K. According to the used method, this effect can be attributed to some uncoupled nuclear spins or change of the existing planar anisotropy accounted for by the *g*-vector components $g_{x}$ and $g_{y}$.

The values of all model parameters of the respective compounds are given in Table 3 and Table 4. In particular, for both molecules the ‘*h*’-field parameters read

$$\begin{array}{cc} \; & h_{1/2}=\frac{E_{0,1/2,m}^{f,(1)}-E_{1,1/2,m}^{f,(1)}}{E_{0,1/2}^{\left(1\right)}-E_{1,1/2}^{\left(1\right)}},\\ \; & h_{3/2}=\frac{E_{1,3/2,m}^{f,(1)}-E_{1,1/2,m}^{f,(1)}}{E_{1,3/2}^{\left(1\right)}}-\frac{E_{0,1/2,m}^{f,(1)}-E_{1,1/2,m}^{f,(1)}}{E_{0,1/2}^{\left(1\right)}-E_{1,1/2}^{\left(1\right)}}, \end{array}$$

where m=±1/2.

### 4.3. Susceptibility in ac Field

**Figure 14:**
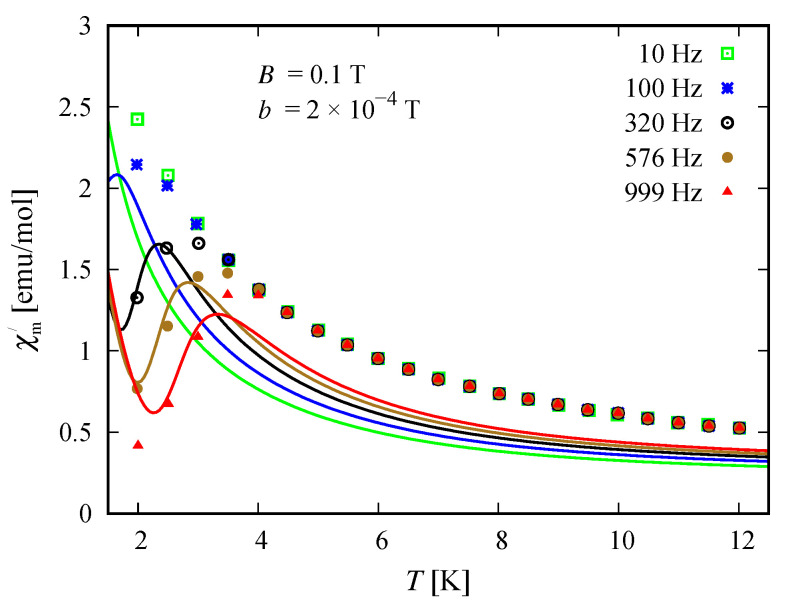
Temperature dependence of the in-phase susceptibility for the reduced compound [(Pc)Er{Pc{N(C${}_{4}$H${}_{9}$)${}_{2}$}${}_{8}$}]${}^{-}$. The symbols and solid lines depict the experimental data from Reference [9] and the theoretical results, respectively. The computations are made with the aid of spin–sigma model (1) and (12). The values of all relevant parameters are provided in Table 5.

**Figure 15:**
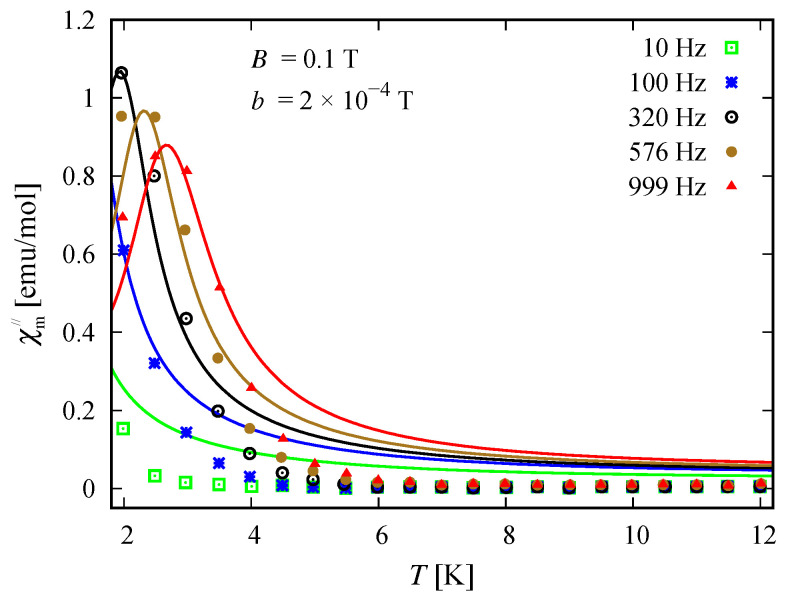
Out-of-phase susceptibility of the reduced compound [(Pc)Er{Pc{N(C${}_{4}$H${}_{9}$)${}_{2}$}${}_{8}$}]${}^{-}$ as a function of absolute temperature. The experimental data provided in Reference [9] are depicted by symbols. The theoretical results are shown by solid lines and are obtained by using (12) and the spin-sigma model (1) with fitting parameters given in Table 5.

Among both compounds, only the reduced member [(Pc)Er{Pc{N(C${}_{4}$H${}_{9}$)${}_{2}$}${}_{8}$}]${}^{-}$ exhibits slow magnetic relaxation. The process is driven by the presence of a dc field and occurs at temperatures lower than 3 K, indicating the existence of a small energy barrier. The corresponding dynamics is well described by the spin–sigma model and the relations (12). A comparison between the experimental data and theoretical calculations is shown in Figure 14 and Figure 15. The values of all fitting parameters are given in Table 5. In particular, the values of both $h_{1/2}=0.7$ and $h_{3/2}=0.7$ parameters predict a small energy barrier, $0.69\;\mathrm{meV}\leq U_{\mathrm{eff}}\leq 0.73\;\mathrm{meV}$. The result is compatible with the experimentally measured energy barrier of approximately 0.73 meV reported in Reference [24]. Nevertheless, the used model does not shed light on the underlying relaxation pathway.

For the unprotonated compound, we have $\tau \to \tau_{0}$ and hence observe no time delay in the response to the alternating field. As a result, from (12), we get $\chi_{m}^{\prime }\equiv \chi_{m}^{}$ and $\chi_{m}^{\prime \prime }=0$.

## 5. Discussion

With the aim of exploring a possible role of the intramolecular exchange mechanism in governing the magnetic properties of Er${}^{3+}$ single ion magnets, we adapt a recently constructed effective spin-like model [25] and study the magnetic properties of rare earth compounds Na${}_{9}$[Er(W${}_{5}$O${}_{18}$)${}_{2}$] and [(Pc)Er{Pc{N(C${}_{4}$H${}_{9}$)${}_{2}$}${}_{8}$}]${}^{\cdot /-}$. To this end, in contrast to the methods working with free-ion basis states and the added perturbative crystal field effect, we considered the 4f unpaired electrons as delocalized occupying molecular orbitals, see Section 2. The theoretical results obtained with the aid of the named model are in good agreement with the experimental findings [9,24].

In particular, calculating the magnetic properties of the single ion magnet Na${}_{9}$[Er(W${}_{5}$O${}_{18}$)${}_{2}$] we use the spin-sigma Hamiltonian (1) and the Heisenberg (3) models. Both models predict a Kramer’s doublet, with $s_{23}=1$, $s=\frac{1}{2}$, $m=\pm \frac{1}{2}$ as a ground state, and suggest the second doublet, characterized by the spin quantum numbers $s_{23}=0$, $s=\frac{1}{2}$, $m=\pm \frac{1}{2}$, as a first excited state. Furthermore, in the energy spectra of both models, the quartet level appears as a second excited level. In this regard, both models describe reasonably well the two ground state transitions with intensities given by (6), (7) and shown in Figure 3 and Figure 4 with solid lines. Since for all *T*, the difference in the values of $p_{0}\left(T\right)$ obtained via the spin–sigma and Heisenberg Hamiltonians is negligible, both models predict the same magnitudes for each integrated intensity, see (6) and (7). Nevertheless, only the energy spectrum (2) accounts for the existence of a third excited level and therefore explains the appearance of the high temperature magnetic excitation. The high scattering probability related to the magnitude of the third peak is reproduced only by accounting for the probability of observing a transition from quartet non-magnetic states such as the spin–quadrupole ones [31,32,33,34,35]. The respective intensity is given by (8). On the other hand, the spectrum (4) does not account for the contribution of electrons’ orbital moments to the system’s energy. Therefore, in the presence of an external magnetic field the Heisenberg model fails to explain the observed dc magnetization and susceptibility measurements. While the spin–sigma model provides a good qualitative and quantitative description of these properties, see Figure 5 and Figure 6. For B>0, it predicts an abrupt transition in the ground state from $s=\frac{1}{2}$ and $m=\pm \frac{1}{2}$ to $s=\frac{3}{2}$ and $m=\frac{3}{2}$ that is stabilized by reaching the saturation of the magnetization above 2 T. Respectively, the total effective magnetic moment changes without exhibiting an intermediate magnetization step. According to the applied method, such shifting of the energy levels and hence variation in the total spin value is driven by unique interaction terms related to the electrons’ orbital moments that enter into the initial Hamiltonian for B≠0, see Reference [25]. The contribution of these interaction terms is effectively accounted for by the ‘*h*’-field parameters with values given in Table 1. The value of the total *g*-factor points to the existence of a planar anisotropy, with $g_{\alpha }\approx g_{\nu }$ and $g_{\alpha },g_{\nu }≲g_{\beta }$ for α≠β≠ν∈{x,y,z}. The dynamic properties of Na${}_{9}$[Er(W${}_{5}$O${}_{18}$)${}_{2}$] are characterized only with the aid of the spin–sigma model. A comparison between the theoretical and experimental results is depicted in Figure 8 and Figure 9. We have a good agreement with the experimental data for both the in-phase and out-of-phase susceptibilities. The calculations yield an energy barrier to magnetization reversal in the interval 3.81–3.94 meV. This result is compatible with the experimentally observed value of 3.8 meV reported in Reference [9].

To explore the magnetic properties of the single ion magnets, [(Pc)Er{Pc{N(C${}_{4}$H${}_{9}$)${}_{2}$}${}_{8}$}]${}^{\cdot /-}$, we apply only the spin–sigma model (1). As we have demonstrated for the other compound, see Figure 5 and Figure 6, Hamiltonian (3) is not suitable for studying the magnetic properties of rare earth complexes. The results obtained with the aid of (1) are in good agreement with the available experimental data provided in Reference [24]. Reproducing the dc magnetization and susceptibility measurements, see Figure 10 and Figure 11, we detected a reversal of the quartet level from excited to ground state level. Accordingly, the total spin changes from $s=\frac{1}{2}$ to $s=\frac{3}{2}$, as is the case with Na${}_{9}$[Er(W${}_{5}$O${}_{18}$)${}_{2}$]. As the theory suggests, the observed transition from partially to fully polarized spin state for low values of the applied field is a consequence of the interaction of the external magnetic field with that intrinsic to the molecule arising from the electrons’ orbitals. This effect is accounted for effectively by the ‘*h*’ parameters with values given in Table 3 and Table 4. Moreover, according to the calculations for the saturation of the magnetization, the neutral compound [(Pc)Er{Pc{N(C${}_{4}$H${}_{9}$)${}_{2}$}${}_{8}$}]${}^{\cdot }$ is characterized by a larger *g*-factor value than that of the reduced compound. On the other hand, the saturation of the magnetization for [(Pc)Er{Pc{N(C${}_{4}$H${}_{9}$)${}_{2}$}${}_{8}$}]${}^{-}$ remains unchanged in the temperature domain 2–6 K. The stabilization of the saturation is related to a small variation of the corresponding *g*-factor value, see Table 4. According to the applied method and model, this effect is either due to a change in the planar anisotropy or from a contribution of uncoupled nuclear spins. The field-induced slow magnetic relaxation in the reduced member is qualitatively well reproduced by the energy spectrum of the spin–sigma model (2). The calculated energy barrier is bounded in the domain $0.69\;\mathrm{meV}\leq U_{\mathrm{eff}}\leq 0.73\;\mathrm{meV}$ and it lies very close to the experimentally observed value of approximately 0.73 meV [24]. The energy difference between the zero and ac field ground states and the second excited energy levels provides the amount of energy that has to be applied to observe a jump in the magnetization. It is accounted for by the ‘*h*’-field parameters with values provided in Table 5 and fitted to the in-phase and out-of-phase susceptibility data depicted in Figure 14 and Figure 15. The absence of a slow magnetic relaxation in the case B=0 and B=0.1 T, demonstrated by the neutral compound [(Pc)Er{Pc{N(C${}_{4}$H${}_{9}$)${}_{2}$}${}_{8}$}]${}^{\cdot }$, leaves the zero field values of both ‘*h*’-field parameters unchanged and suggests the absence of a preferential easy plain or axis, that is, $g_{\alpha }\approx g_{\beta }$ for all α≠β∈{x,y,z}. A slow magnetic relaxation may be observed for higher magnitudes of a dc or a ac generated field. Therefore, above 2 K the in-phase susceptibility describes the typical paramagnetic-like behavior, which is trivially reproduced. Accordingly, in the all-temperature domain, the out-of-phase susceptibility equals zero.

## Figures and Tables

**Table 1:** The values of all parameters entering in (2) for Na${}_{9}$[Er(W${}_{5}$O${}_{18}$)${}_{2}$]. The first row shows the temperature corresponding to the measurements. The evaluation of J and the spectroscopic parameters ‘*c*’ is made in accordance to the neutron spectroscopy data shown in Figure 3 and Figure 4. The ‘*h*’ parameters are fitted in with respect to the magnetization measurements depicted in Figure 5. All experimental data are taken from Reference [9].

*T* [K]	5	5	5	5	50	2	2	2
	J [meV]	*c* ${}_{23,1}^{1,1/2}$	*c* ${}_{23,1}^{1,3/2}$	*c* ${}_{23,1}^{0,1/2}$	*c* ${}_{23,2}^{0,1/2}$	*h* ${}_{1/2}$	*h* ${}_{3/2}$	*g*
B=0 T	2.41(6)	1	1	−0.6	−3.54(3)	100	10	—
B=0.1 T	2.41 (6)	1	1	−0.6	−3.54(3)	0.85	−0.92	2.92
B>3 T	2.41(6)	1	1	−0.6	−3.54(3)	0.15	−0.35	2.92

**Table 2:** Values of the ‘*h*’-field and all ac field parameters entering (12) used to describe the ac susceptibility behavior of the compound Na${}_{9}$[Er(W${}_{5}$O${}_{18}$)${}_{2}$] depicted in Figure 8 and Figure 9. The first column lists the frequencies of the alternating magnetic field at which all parameters are fitted.

ω [Hz]	$a_{1}$	$a_{2}$	$d_{1}$	$d_{2}$	$u_{1}$	$u_{2}$	$c_{1}$	$c_{2}$	*h* ${}_{1/2}$	*h* ${}_{3/2}$
50	1.07	0.33	0.37	0.6	0.16	0.075	0.6	0.075	−0.09	−0.05
100	1.13	0.3	0.28	0.55	0.16	0.075	0.6	0.075	−0.09	−0.05
250	1.28	0.32	0.25	0.45	0.16	0.075	0.6	0.075	−0.09	−0.05
500	1.3	0.32	0.15	0.3	0.16	0.075	0.6	0.075	−0.09	−0.05
1500	1.5	0.38	0.15	0.1	0.16	0.075	0.6	0.075	−0.09	−0.05

**Table 3:** The values of all parameters entering (2) for the neutral compound [(Pc)Er{Pc{N(C${}_{4}$H${}_{9}$)${}_{2}$}${}_{8}$}]${}^{\cdot }$. The first row shows the temperature under which a parameter is fitted. The evaluation of J, the spectroscopic ‘*c*’ and field ‘*h*’ parameters is made with respect to the magnetization measurements depicted on Figure 10. The experimental data are provided in Reference [24].

*T* [K]	2–300	2–300	2–300	2–300	—	2–6	2–6	2–6
	J [meV]	*c* ${}_{23,1}^{1,1/2}$	*c* ${}_{23,1}^{1,3/2}$	*c* ${}_{23,1}^{0,1/2}$	*c* ${}_{23,2}^{0,1/2}$	*h* ${}_{1/2}$	*h* ${}_{3/2}$	*g*
B=0 T	2.36	1	1	−0.23	—	1	1	—
B>3 T	2.36	1	1	−0.23	—	0.1	−0.7	3.38

**Table 4:** List of parameters’ values entering (2) in the case of the compound [(Pc)Er{Pc{N(C${}_{4}$H${}_{9}$)${}_{2}$}${}_{8}$}]${}^{-}$. The first row shows the temperature range according to which the corresponding values are obtained. The exchange J, spectroscopic ‘*c*’ and field ‘*h*’ parameters are fitted with respect to the magnetization data taken from Reference [24] and depicted in Figure 10. Here, k=1,3.

*T* [K]	2–300	2–300	2–300	—	2–6	2–6	2–6
	J [meV]	*c* ${}_{23,1}^{1,k/2}$	*c* ${}_{23,1}^{0,1/2}$	*c* ${}_{23,2}^{0,1/2}$	*h* ${}_{1/2}$	*h* ${}_{3/2}$	*g*
B=0 T	1.56	1	0.23	—	1	1	—
B>3 T	1.56	1	0.23	—	0.1	−0.7	3−3.15

**Table 5:** Values of all fitting parameters entering (12) and the ‘*h*’-field ones used to reproduce the ac susceptibility data of the compound [(Pc)Er{Pc{N(C${}_{4}$H${}_{9}$)${}_{2}$}${}_{8}$}]${}^{-}$ shown on Figure 14 and Figure 15. The first column includes the frequency of the alternating magnetic field according to which the corresponding values are obtained.

ω [Hz]	$a_{1}$	$a_{2}$	$d_{1}$	$d_{2}$	$u_{1}$	$u_{2}$	$c_{1}$	$c_{2}$	*h* ${}_{1/2}$	*h* ${}_{3/2}$
10	0.4	0.01	0.01	0.51	0.17	0.11	1	0.11	0.7	0.7
100	1	0.4	0.01	0.5	0.17	0.11	1	0.11	0.7	0.7
320	1.45	0.48	0.01	0.488	0.17	0.11	1	0.11	0.7	0.7
576	1.76	0.59	0.01	0.257	0.17	0.11	1	0.11	0.7	0.7
999	2.05	0.73	0.01	0.01	0.17	0.11	1	0.11	0.7	0.7

## Data Availability

The data generated within this research is included in the paper.
